# The recurrence risk of gestational diabetes according to the number of abnormal values in the oral glucose tolerance test

**DOI:** 10.1111/aogs.15148

**Published:** 2025-05-02

**Authors:** Jenni Pukkila, Marja Vääräsmäki, Sanna Eteläinen, Sanna Mustaniemi, Hilkka Nikkinen, Mika Gissler, Tuija Männistö, Hannele Laivuori, Eero Kajantie, Elina Keikkala, the FinnGeDi Study Group

**Affiliations:** ^1^ Research Unit of Clinical Medicine, Department of Obstetrics and Gynecology, Medical Research Center, Oulu University Hospital University of Oulu Oulu Finland; ^2^ Welfare Epidemiology and Monitoring Unit, Department of Public Health Finnish Institute for Health and Welfare Helsinki and Oulu Finland; ^3^ Datasets and Dataproducts Unit, Department of Data and Analytics Finnish Institute for Health and Welfare Helsinki Finland; ^4^ Department of Molecular Medicine and Surgery Karolinska Institute Stockholm Sweden; ^5^ Academic Primary Health Care Centre Stockholm Sweden; ^6^ Department of Clinical Chemistry, Institute of Clinical Medicine University of Eastern Finland Kuopio Finland; ^7^ Joint County Authority for ISLAB Laboratories Kuopio Finland; ^8^ Translational Medicine Research Unit University of Oulu Oulu Finland; ^9^ Faculty of Medicine and Health Technology, Center for Child, Adolescent and Maternal Health Research Tampere University Tampere Finland; ^10^ Department of Obstetrics and Gynecology, Tampere University Hospital Wellbeing Services County of Pirkanmaa Tampere Finland; ^11^ Medical and Clinical Genetics University of Helsinki and Helsinki University Hospital Helsinki Finland; ^12^ Helsinki Institute of Life Science, Institute for Molecular Medicine Finland University of Helsinki Helsinki Finland; ^13^ Children's Hospital University of Helsinki and Helsinki University Hospital Helsinki Finland; ^14^ Department of Clinical and Molecular Medicine Norwegian University of Science and Technology Trondheim Norway

**Keywords:** diabetes, high‐risk pregnancy, pregnancy, women's health issues

## Abstract

**Introduction:**

Oral glucose tolerance test (OGTT) results may be used to estimate the risk of recurrent gestational diabetes mellitus (GDM) in a subsequent pregnancy in the different study settings. This study assesses the association between the number of abnormal glucose values in the OGTT in the first pregnancy and GDM recurrence in a subsequent pregnancy in a Nordic cohort.

**Material and Methods:**

This register‐based cohort study included 1677 women who had their first singleton delivery in 2009, underwent a 75 g 2‐h OGTT during the pregnancy, and gave birth at least once more within 10 years according to the Finnish Medical Birth Register. The cut‐off values were as follows: ≥5.3 mmol/L at fasting, ≥10.0 mmol/L at 1 h, and ≥8.6 mmol/L at 2 h. The odds ratio (OR) for GDM recurrence in the second pregnancy was analyzed via multivariable logistic regression adjusted for other potential factors associated with recurrence risk.

**Results:**

During the first pregnancy, GDM was diagnosed in 331 (24.5%) women based on one (*n* = 250) or two or three (*n* = 81) abnormal glucose values in the OGTT. The total recurrence rate for GDM in the subsequent pregnancy was 56.2%. The rate differed significantly between women with one (51.6%) and women with two or three (70.4%) abnormal values in first‐pregnancy OGTT. Compared with those with normal OGTT results, the adjusted OR (aOR) for GDM in the subsequent pregnancy in women with one abnormal glucose value was 6.00 (95% CI, 4.34–8.30), while it was 13.37 (7.52–23.76) in women with two or three abnormal values. The odds for GDM recurrence among those with two or three abnormal glucose values was double compared to those with only one abnormal value (aOR 2.03, 1.12–3.68).

**Conclusions:**

Primiparous women with one abnormal glucose value in the first OGTT have remarkable odds of GDM recurrence, with the odds doubling when there are two or three abnormal values during the first pregnancy. These findings can be used when planning effective counseling, prevention, and screening strategies for GDM in the subsequent pregnancy.

## Graphical abstract

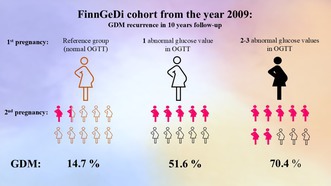

**Abbreviations**
BMI: body mass index
CCG: current care guidelines
DM: diabetes mellitus
GDM: gestational diabetes mellitus
gw: weeks of gestation
IADPSG: International Association of Diabetes and Pregnancy Study Groups
IQR: interquartile range
LGA: large for gestational age
MBR: Medical Birth Register
OGTT: oral glucose tolerance test
SES: socioeconomic status
T1D: type 1 diabetes
T2D: type 2 diabetes

**Key message**

Two or three abnormal glucose values in the oral glucose tolerance test in the first pregnancy are associated with double the odds of gestational diabetes in the subsequent pregnancy compared with one value, with the overall recurrence rate reaching 56%.

## 1 INTRODUCTION

The World Health Organization defines gestational diabetes mellitus (GDM) as elevated blood glucose levels observed for the first time during pregnancy.^1^ Worldwide, its prevalence varies between 2% and 25%, depending mainly on the screening strategy.^2^ The risk of GDM recurrence in a subsequent pregnancy varies between 40% and 60%^3^, ^4^ and appears to be multifactorial. GDM and type 2 diabetes (T2D) share a partially similar genetic background, but different genetic risk genes are prevalent in different populations. This is one reason underlying the association between ethnicity and the risk of GDM as well as its recurrence.^2^, ^5^, ^6^ Obesity and advanced age are reported to be the major clinical risk factors that contribute to GDM recurrence.^3^, ^7^ Other clinical factors such as multiparity, high weight gain between pregnancies, and very long or very short interdelivery intervals are related to a higher risk of recurrence. In addition, severe GDM, characterized by a need for insulin treatment or the delivery of a macrosomic newborn, appears to recur more frequently than GDM without these conditions.^3^, ^8^

The oral glucose tolerance test (OGTT) is a standard approach for the screening and diagnosis of GDM. However, various recommendations have been proposed regarding the number of glucose measurements, the cut‐offs, and the number of abnormal OGTT values required for a diagnosis of GDM.^1^, ^9^, ^10^, ^11^, ^12^, ^13^ An OGTT typically includes glucose concentrations measured at fasting and 1 and 2 h after the oral loading of 75 g of liquid glucose.^10^ Several studies have reported that a higher number of abnormal glucose values in OGTT appears to be associated with higher risks of both GDM recurrence and later T2D,^14^, ^15^, ^16^, ^17^ whereas one study observed that especially an abnormal 2‐h afterload glucose increase was associated with a higher GDM recurrence risk.^18^

Against this background, the aim of this study is to assess the association between the number of abnormal OGTT glucose values in the first pregnancy and the odds of GDM recurrence in subsequent pregnancies. We hypothesize that women with two or three abnormal OGTT values are at a higher odds of GDM recurrence compared to those with only one abnormal value.

## 2 MATERIAL AND METHODS

### 2.1 Study design and participants

**FIGURE 1:**
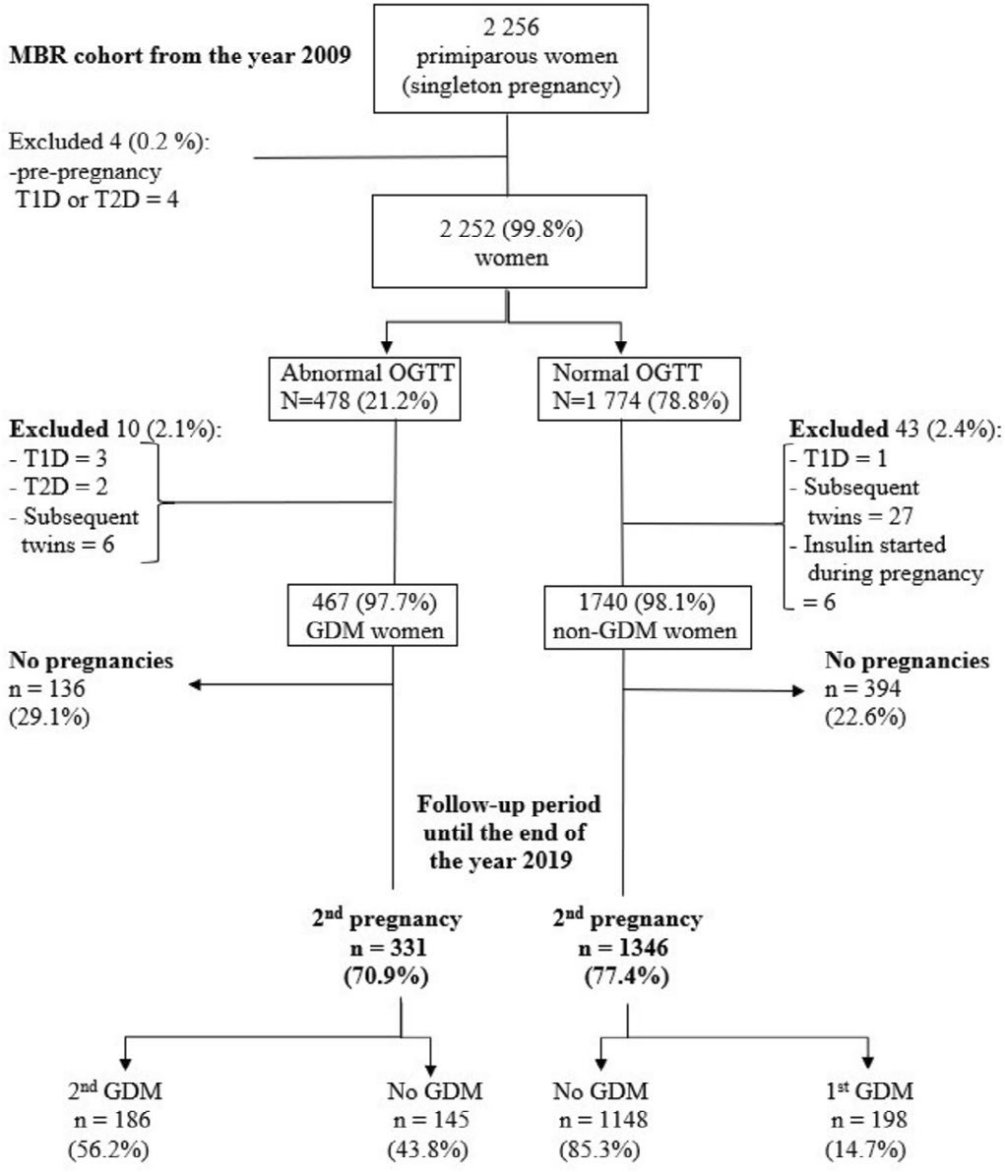
Flow chart of primiparous women with available quantitative data on oral glucose tolerance test (OGTT) results of women who delivered in 2009 with follow up until the end of 2019. DM, diabetes mellitus; GDM, gestational diabetes; MBR, Medical Birth Register; OGTT, oral glucose tolerance test; T1D, type 1 diabetes; T2D, type 2 diabetes.

The register‐based Finnish Gestational Diabetes (FinnGeDi) study is based on a longitudinal cohort that included all women with singleton pregnancies who delivered in Finland in 2009 according to the Finnish Medical Birth Register (MBR). The cohort data have been reported in detail elsewhere.^19^ The MBR included data on GDM status, background characteristics including maternal age, pre‐pregnancy body mass index (BMI), socioeconomic status (SES), diagnostic codes during pregnancy and delivery according to the International Classification of Diseases' (ICD) 10th revision, and detailed perinatal and neonatal data. For this study, numerical OGTT results including all three measured glucose values for 2254 pregnant primiparous women were obtained from six hospital laboratory databases (tertiary‐level hospitals in Oulu and Tampere and secondary‐level hospitals in Kajaani, Lappeenranta, Pori, and Seinäjoki). The data were combined with the MBR data using personal identity codes in a secure computing environment at the Finnish Institute for Health and Welfare. Women with pre‐pregnancy diabetes mellitus (DM; type 1 or type 2; ICD‐code E10‐E11, E13‐E14, or O24.0‐O24.3) were excluded. The possibility of subsequent pregnancy was surveyed in the MBR until the end of 2019. Women who did not have a subsequent pregnancy, who were diagnosed with pre‐pregnancy DM before the subsequent pregnancy, or who experienced multifetal pregnancy were excluded. The study population included 2207 primiparous women with OGTT performed during pregnancy, of whom 1677 (76.0%) had at least one subsequent pregnancy within the follow‐up period and were classified according to the number of abnormal values. Of them, the first‐pregnancy OGTT value was abnormal in 331 (19.7%) women (one abnormal value: *n* = 250, 75.5%; two or three abnormal values: *n* = 81, 24.5%), who were thus diagnosed with GDM, whereas the OGTT values were normal in 1346 (80.3%) women (reference group) (Figure 1).

### 2.2 Current care guidelines

Based on the Finnish current care guidelines (CCG), the 2 h 75 g OGTT cut‐off values for GDM were as follows: plasma glucose concentration ≥5.3 mmol/L at fasting, ≥10.0 mmol/L at 1 h, and ≥8.6 mmol/L at 2 h.^1^, ^20^ GDM was diagnosed if at least one value exceeded the cutoff.^1^, ^20^ If the fasting glucose value was indicative of diabetes (≥7 mmol/L), the OGTT was discontinued, and further examinations were carried out in the maternity outpatient clinic. In the other cases, the test was completed. The OGTT was recommended for all women at 24–28 weeks of gestation (gw), except those at a very low risk of GDM (primiparous women under 25 years with BMI <25 kg/m^2^ and multiparous women under 40 years with BMI <25 kg/m^2^ and without previous macrosomia or T2D in a first‐degree relative). In high‐risk women (prior GDM, pre‐pregnancy BMI >35 kg/m^2^, glucosuria in early pregnancy, T2D in a first‐degree relative, oral glucocorticoid treatment, or polycystic ovary syndrome), the OGTT was performed at 12–16 gw and, if normal, repeated at 24–28 gw. An additional OGTT was performed upon clinical suspicion of GDM. All women with GDM received interventions to promote a healthy lifestyle, including a healthy diet and physical activity, and started to self‐monitor their capillary glucose. Pharmacological treatment (insulin and/or metformin) was initiated if the fasting values in the self‐monitoring repeatedly exceeded 5.5 mmol/L or if the 1 h postprandial value exceeded 7.8 mmol/L, despite the aforementioned interventions. The screening policy and cutoffs remained similar during the follow‐up period.^1^, ^20^ Before the published CCG in 2008, a risk‐factor‐based GDM screening was used.

### 2.3 Definitions

All maternal and neonatal parameters, except for the glucose values in the OGTT, are based on the MBR. Maternal age was reported based on years at the time of delivery. Weight (kg) and height (cm) were self‐reported measurements at the first maternity clinic visit. Pre‐pregnancy BMI was calculated (kg/m^2^) and categorized as under‐ or normal weight (<25.0 kg/m^2^), overweight (25–29.9 kg/m^2^), or obese (≥30 kg/m^2^). Changes in weight and BMI between the first and second pregnancies were calculated based on MBR data regarding pre‐pregnancy weight and height reported in the first and second pregnancies. BMI changes between pregnancies were categorized as follows: <−4, −4 to −2.01, −2 to 2, 2.01 to 4, and >4 (where −2 to 2 was set as a reference range). Maternal SES was assessed based on maternal occupational status at first pregnancy and further classified into four categories: upper‐level clerical (upper and lower university degree education, administrative, managerial, professional, and related occupations), lower‐level clerical (university of applied sciences, college, administrative, and clerical occupations requiring higher education), manual workers (e.g., blue‐collar, agricultural, distribution, and service workers), and others (e.g., self‐employed, students, pensioners, unemployed, stay‐at‐home mothers, and others with an unclassified status). SES data were missing for 505 (22.8%) women. Infant birthweight standard deviation (SD) was calculated according to the Finnish growth charts (standardized according to sex, gestational age, parity, and number of fetuses) created by Sankilampi et al.^21^ Large for gestational age (LGA) was defined as birthweight >+2 SD. The interdelivery interval was calculated between the dates of the first and second deliveries and further categorized as ≤24 or >24 months.

Glucose concentrations and the dates of OGTT performance were based on the laboratory database. If the OGTT was performed more than once during the first pregnancy, we used the first abnormal glucose value. If the OGTT was repeatedly normal, the first result was used. Early OGTT was defined as OGTT performed at ≤20 gw, with a positive result in such a test being defined as early GDM. The number of abnormal glucose values (zero to three) was classified and reported as follows: zero abnormal values as a normal OGTT (reference group), one abnormal glucose value, and two or three abnormal glucose values based on the CCG cutoffs detailed above.^1^, ^20^ Data concerning pharmacological treatment initiation for GDM were obtained from the MBR, where the variable “insulin started during pregnancy” was reported for the entire follow‐up period, and “other pharmacological treatment started” (metformin in practice) was reported from 2016 onwards. Information about whether OGTT was performed in the second pregnancy was also obtained from the MBR.

### 2.4 Outcome

The primary outcome was the GDM status in the subsequent pregnancy. GDM was diagnosed if at least one of the following criteria based on the MBR was met: ICD‐10 code of GDM (O24.4 or O24.9) was recorded, OGTT was described as “abnormal,” or insulin or another pharmacological treatment for GDM was started during the pregnancy.

### 2.5 Statistical methods

IBM SPSS 29.0 software was used to perform statistical analyses. Differences between continuous variables were analyzed using Student's *t*‐test for normally distributed parameters and Mann–Whitney's *U*‐test for parameters with skewed distributions. The distributions were visually assessed using histograms. Categorical data were analyzed using Pearson's chi‐squared test or Fisher's exact test in the case of a small sample size. Continuous data are presented as the mean and standard deviation or the median and interquartile range (IQR). Categorical data are reported as numbers and percentages (%).

Univariate logistic regression was used to analyze the associations between different variables and the outcome. Multivariable logistic regression was used to analyze the association between the number of abnormal OGTT values and the likelihood of developing recurrent GDM by adjusting for the following variables: maternal age and pre‐pregnancy BMI at the second delivery, pharmacological treatment and LGA in the first pregnancy, interdelivery interval, and pre‐pregnancy BMI change between pregnancies. The results are presented as crude and adjusted odds ratios (ORs) and 95% confidence intervals (CIs).

## 3 RESULTS

### 3.1 Characteristics in the first pregnancy

In total, 2252 primiparous women with numerical OGTT results collected from the six delivery‐hospital databases were identified from the MBR. Four women with pre‐pregnancy DM diagnosed before the first delivery, six women with pre‐pregnancy DM before the subsequent pregnancy, six women who started insulin treatment despite normal OGTT results without a pre‐pregnancy DM diagnosis in the first pregnancy, and 33 women with multifetal pregnancy in the subsequent pregnancy were excluded. After exclusion, at least one subsequent pregnancy was observed in 1677 women within the follow‐up period. These women formed the final population for this study (Figure 1). Abnormal OGTT results were observed for 331 women (19.7%). Of those, 250 women (75.5%) had one abnormal glucose value, and 81 women (24.5%) had two or three abnormal values. Sixty‐five (19.6%) had two, and 16 (4.8%) had three abnormal OGTT values. The reference group consisted of 1346 women (80.3%) with normal OGTT results in their first pregnancy. When comparing maternal characteristics in the GDM groups to those of the reference group, the only significant differences were higher BMI and a higher frequency of obesity in the women with GDM in both their first and second pregnancies (Table 1).

**TABLE 1:** Maternal and perinatal characteristics in the first pregnancy and maternal characteristics in the second pregnancy according to oral glucose tolerance test (OGTT) status.

	One value abnormal			Two or three values abnormal			*p* value	Reference group	
	*N* = 250		*p* value	*N* = 81		*p* value		*N* = 1346	
	Mean/*N*	SD/(%)	One value abnormal versus reference group	Mean/*N*	SD/(%)	Two or three values abnormal versus reference group	Two or three values abnormal versus one abnormal value	Mean/*N*	SD/(%)
Maternal and perinatal characteristics in the 1st pregnancy									
Early GDM (≤20 gw)	23	(9.2)	<0.001 F	7	(8.6)	<0.001 F	1.000 F	0	(0.0)
Numeric OGTT values, mmol/L									
Fasting value	5.0	0.5	<0.001	5.4	0.6	<0.001	<0.001	4.6	0.3
1‐h value	9.1	1.7	<0.001	11.1	1.2	<0.001	<0.001	7.1	1.4
2‐h value	6.8	1.3	<0.001	8.5	1.9	<0.001	<0.001	5.7	1.1
Pharmacological treatment started	13	(5.2)	<0.001 F	7	(8.6)	<0.001	0.284	0	(0.0)
Age, years	28.1	4.6	0.247	27.8	4.8	0.825	0.688	27.7	4.4
Pre‐pregnancy BMI, kg/m^2^	26.8	5.6	<0.001	28.8	5.7	<0.001	0.004	25.4	4.5
<25.0	111	(44.6)		21	(26.3)			699	(52.4)
≥25	138	(55.4)		59	(73.8)			635	(47.6)
25–29.9	69	(27.7)		30	(37.5)			451	(33.8)
≥30	69	(27.7)		29	(36.3)			184	(13.8)
Socioeconomic status			0.091			0.357	0.996		
Upper‐level clerical	36	(14.4)		11	(13.6)			243	(18.1)
Lover‐level clerical	77	(39.7)		23	(28.4)			410	(30.5)
Manual workers	39	(21.1)		13	(16.0)			143	(10.5)
Others	42	(21.6)		13	(16.0)			247	(18.4)
Missing	56	(22.4)		21	(25.9)			303	(22.5)
Birthweight, g	3498	475	0.817	3456	730	0.413	0.630	3506	519
Birthweight standard deviation (SD)^20^	0.10	1.0	0.081	0.20	1.37	0.092	0.533	0.003	1.01
LGA (>2 SDs)	3	(1.2)	0.345 F	8	(9.9)	0.001	0.001	31	(2.3)
Maternal characteristics in the 2nd pregnancy									
OGTT performed	232	(92.8)	<0.001 F	74	(91.4)	<0.001 F	0.809	851	(63.2)
GDM	129	(51.6)	<0.001	57	(70.4)	<0.001 F	0.003 F	198	(14.7)
Age, years	30.9	4.9	0.317	30.9	4.9	0.551	0.987	30.6	4.5
Pre‐pregnancy BMI, kg/m^2^	27.6	6.1	0.002	29.4	6.2	<0.001	0.021	26.3	5.3
<25	101	(40.9)		20	(24.7)			630	(47.4)
≥ 25	146	(59.1)		61	(75.3)			699	(52.6)
25–29.9	69	(27.9)		29	(35.8)			408	(30.7)
≥ 30	77	(31.2)		32	(39.5)			291	(21.9)
Weight change between pregnancies, kg	2.4	6.6	0.520	1.9	8.5	0.318	0.643	2.7	6.6
BMI change, kg/m^2^	0.87	2.4	0.512	0.66	3.0	0.273	0.580	0.98	2.5
Classified change			0.114			0.007	0.362		
>4	19	(7.7)		8	(10.0)			119	(9.0)
2.01 to 4	47	(19.1)		15	(18.8)			217	(16.5)
−2 to 2	155	(63.0)		45	(56.3)			901	(68.4)
−2.01 to −4	20	(8.1)		7	(8.8)			62	(4.7)
<−4	5	(2.0)		5	(6.3)			18	(1.4)
Interdelivery interval, years, median, IQR	2.4	1.8; 3.2	0.496	2.7	1.8; 3.5	0.374	0.243	2.5	1.8; 3.4
≤24 months	83	(33.2)	0.883	24	(29.6)	0.627 F	0.587 F	438	(32.5)
>24 months	167	(66.8)		57	(70.4)			908	(67.5)

*Note*: The reference group consisted of those with a normal OGTT result in the first pregnancy. Criteria from Sankilampi et al.^21^ Analyses were performed by Student's *t*‐test, chi‐squared test, or Fisher's exact test (*F*), and values are presented as mean and standard deviation (SD), and *n* (%). Time between pregnancies was analyzed using Mann–Whitney's *U*‐test and values are presented as median and interquartile range (IQR). Upper‐level clerical: upper and lower university degree education, administrative, managerial, professional and related occupations; lower‐level clerical: university of applied sciences, college, administrative and clerical occupations requiring higher education; manual workers: for example, blue‐collar worker, agricultural worker, distribution worker, service worker and equivalents, and others (e.g., self‐employed, students, pensioners, unemployed, stay‐at‐home mothers, and women with an unclassified status). Missing values: pre‐pregnancy BMI in the first pregnancy: one abnormal glucose value *n* = 1 (0.4%), two or three abnormal values *n* = 1 (1.2%); and in reference group *n* = 13 (1.0%); pre‐pregnancy BMI in the second pregnancy: one abnormal glucose value *n* = 3 (1.2%), reference group *n* = 17 (1.3%); weight and BMI change: one abnormal glucose value *n* = 4 (1.6%), two or three abnormal values *n* = 1 (1.2%), reference group *n* = 30 (2.2%).

Abbreviations: BMI, body mass index; GDM, gestational diabetes; gw, gestational weeks; IQR, interquartile range; LGA, large for gestational age; OGTT, oral glucose tolerance test; SD, standard deviation.

The women with two or three abnormal OGTT values had higher BMI (28.8 kg/m^2^, SD 5.7, kg/m^2^ vs. 26.8 kg/m^2^ SD 5.6 kg/m^2^; *p* = 0.004) and were more frequently overweight or obese (73.8% vs. 55.4%; *p* < 0.001) than women with one abnormal value during the first pregnancy (Table 1).

The frequency of early GDM or the need for pharmacological treatment did not differ between the GDM groups. The mean glucose concentrations in the OGTT at all three time points were higher in women with two or three abnormal glucose values than in women with one abnormal value. The mean birthweight of the newborns did not differ between the study groups, but LGA was more common in women with two or three abnormal values compared to those with one abnormal value (9.9% vs. 1.2%, *p* < 0.001) (Table 1).

### 3.2 Interdelivery characteristics

The mean weight increased by 1.9–2.4 kg and the mean BMI by 0.66–0.98 kg/m^2^ between the pregnancies in all studied groups, with no statistically significant differences between the groups. However, when BMI changes were analyzed in the different categories, a lower proportion of women with two or three abnormal values had a similar BMI between pregnancies (BMI change between pregnancies −2 to 2 BMI units), compared with the reference group (56.3% vs. 68.4%; *p* = 0.008). Of them, 15.1% lost weight equivalent to more than 2 BMI units compared to 6.1% in the reference group. The median interdelivery intervals were 2.4–2.7 years, and 29.6% to 33.2% of women had a subsequent birth before 24 months, indicating no statistically significant differences between the groups (Table 1).

### 3.3 GDM in the second pregnancy

Among women with GDM (*n* = 331) in the first pregnancy, GDM recurred in 186 (56.2%) of them during the second pregnancy. For those 250 women with one abnormal value in the first pregnancy, OGTT was performed in 232 (92.8%) of women in the subsequent pregnancy, and GDM was diagnosed in 129 (51.6%) of these women. Furthermore, for 81 women with two or three abnormal values in the first pregnancy, the corresponding rates were 74 (91.4%) and 57 (70.4%), respectively. Hence, OGTT was performed at equivalent rates in the two GDM groups, but GDM recurrence was more common in women with two or three abnormal OGTT values in the first pregnancy (*p* = 0.003). Of the 1346 women in the reference group, 851 (63.2%) underwent an OGTT in the subsequent pregnancy, and 198 (14.7%) were diagnosed with GDM (Table 1).

In univariate analyses, GDM diagnosis, a higher number of abnormal OGTT values, higher pre‐pregnancy BMI in either of the pregnancies, pharmacological treatment of GDM, macrosomia in the first pregnancy, large weight gain between pregnancies, and advanced maternal age in the second pregnancy were associated with GDM in the subsequent pregnancy (Table S1). In the univariate subanalyses including only women with abnormal values in the OGTT in the first pregnancy, the association with GDM recurrence was observed if there was a higher number of abnormal OGTT values, an abnormal fasting glucose value, a significant increase in BMI between pregnancies, and higher pre‐pregnancy BMI in the first or second pregnancy (Table S2).

**FIGURE 2:**
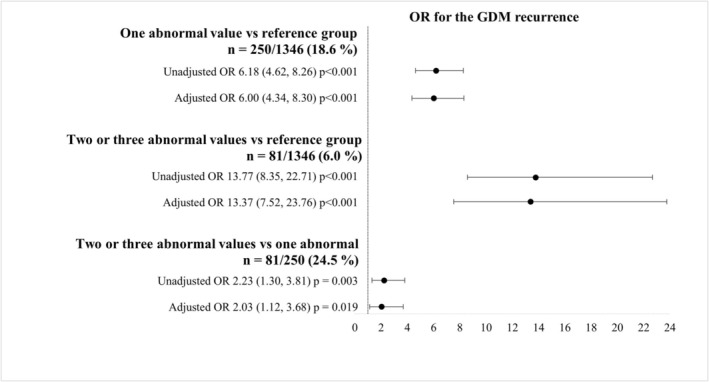
Recurrence of gestational diabetes (GDM) according to the number of abnormal oral glucose tolerance test (OGTT) values in the first pregnancy. The reference group consisted of women with a normal OGTT result in the first pregnancy. Analyses were performed using logistic regression and adjusted for maternal age and body mass index (BMI) at the second pregnancy, insulin or metformin use in the first pregnancy (yes/no), and large for gestational age (yes/no) in the first pregnancy (defined as birthweight >+2 standard deviations),^21^ interdelivery interval (≤24 or >24 months), and BMI change (categorized as <−4, −4 to −2.01, −2 to 2, 2.01 to 4, >4 kg/m^2^). Missing covariates: One abnormal value versus the reference group: *n* = 34 (2.0%); two or three abnormal values versus the reference group *n* = 30 (2.1%); two or three abnormal values versus one abnormal value *n* = 5 (1.5%). Bars represent odds ratios (ORs) and 95% confidence intervals.

When using the reference group for comparison, the women with one abnormal OGTT value in the first pregnancy, the OR for GDM in the second pregnancy was 6.18 (95% CI, 4.62–8.26), and the adjusted OR (aOR) was 6.00 (4.34–8.30) after adjusting for maternal age and pre‐pregnancy BMI at the second delivery, pharmacological treatment, and LGA in the first pregnancy, interdelivery interval, and pre‐pregnancy BMI change between pregnancies. In cases where there were two or three abnormal glucose values in OGTT during the first pregnancy, the OR was 13.77 (8.35–22.71, *p* < 0.001), and the aOR was 13.37 (7.52–23.76) when compared to the reference group. Women with two or three abnormal glucose values had an OR of 2.23 (1.30–3.81) and an aOR of 2.03 (1.12–3.68) for the recurrence of GDM compared with those with only one abnormal glucose value (Figure 2).

## 4 DISCUSSION

In this study based on a Finnish population, GDM in the first pregnancy recurred in more than half of the women (56.2%) in the second pregnancy. The rate of GDM recurrence was 51.6% if one OGTT value was abnormal at the first pregnancy and 70.4% if two or three values were abnormal. Women with two or three abnormal OGTT values had double the odds of recurrence when compared with those with one abnormal OGTT value. Meanwhile, women with one abnormal OGTT value in the first pregnancy had six times higher odds of GDM recurrence when compared to the reference group. These findings remained unchanged after adjusting for several risk factors, such as maternal age, pre‐pregnancy BMI, the severity of prior GDM, changes in BMI between pregnancies, and the amount of time that elapsed between pregnancies.

These findings are consistent with the results observed in East Asian populations, which showed a twofold risk increase in the recurrence when a higher number of abnormal OGTT values were observed in the first pregnancy in China and Japan.^14^, ^15^ In addition, several studies in multiethnic settings have demonstrated that an increased risk of GDM recurrence is associated with higher glucose concentrations or different combinations of abnormal fasting and postprandial values or one abnormal postprandial value.^8^, ^14^, ^15^, ^17^, ^18^, ^22^ Our study suggests that two or three abnormal glucose values in the OGTT can be considered a significant risk factor for recurrent GDM in the Nordic population as well.

As expected, GDM recurrence was strongly associated with previous GDM and a higher number of abnormal OGTT values in the first pregnancy. Pre‐pregnancy overweight and obesity, the need for pharmacological treatment for GDM, and macrosomia in the first pregnancy, interpregnancy weight gain, and advanced maternal age in the second pregnancy were also associated with GDM recurrence. However, maternal age in the first pregnancy, interpregnancy weight loss, and interpregnancy interval were not associated with GDM recurrence. We found that a higher number of abnormal values in an OGTT was associated with increased odds of GDM recurrence, regardless of the potential confounding factors. The incidence of GDM recurrence was higher in the group of two or three abnormal values (70%) compared to the earlier reported general incidence (40%–60%) of GDM recurrence.^3^, ^4^ These women with two or three abnormal values appear to be at higher risk of developing subsequent GDM even though they did not receive more pharmacological treatment or have an earlier diagnosis of GDM compared to women with one abnormal value. Underlying reasons for this finding cannot be explained by our study, but, for example, genetic predispositions or lifestyle factors may be relevant.^2^, ^5^, ^6^ However, our finding highlights the importance of a higher number of abnormal glucose values in the OGTT besides other known risk factors, such as abnormal fasting glucose values, high pre‐pregnancy BMI, and weight gain between pregnancies when evaluating those who are at risk of GDM recurrence.

Women with GDM have a higher BMI, which is typically related to stronger insulin resistance. However, the need to initiate pharmacological treatment was similar in participants with one abnormal OGTT value and in those with two or three abnormal values, reflecting the fact that normoglycemia was achieved with lifestyle interventions as frequently in both groups. Nonetheless, LGA was more common in women with two or three abnormal values compared to those with only one. This may reflect more severe disease or be a consequence of other factors, such as maternal obesity, which is known to be associated with both increased fetal weight and GDM. However, maternal lipid metabolism, which we were not able to measure, might have an effect on LGA regardless of the maternal weight status.^23^, ^24^ These risk factors (higher maternal BMI, pharmacological treatment for the first incidence of GDM, and LGA) were also associated with GDM recurrence in this study, but the effect of a higher number of abnormal OGTT values appeared to outweigh these factors.

Weight changes between the pregnancies were similar in all studied groups, with the mean BMI increase ranging from 0.66 to 0.98 kg/m^2^. A greater proportion of women with GDM showed a reduction in BMI compared to women in the reference group. This could be explained by women with GDM receiving lifestyle interventions,^1^ which have been reported to restrain gestational weight gain^25^ and could lead to equivalency in the weight change between pregnancies when compared to the reference group. Although weight loss has been described as a protective factor against GDM recurrence,^3^ no such association was found in our study, most likely because of the small sample size. In contrast, excessive weight gain between pregnancies is known to increase the risk of GDM recurrence.^3^ An association between higher weight change and GDM recurrence was also found in this study. As in the case of weight change, maternal age was also similar in both pregnancies in all studied groups. Furthermore, maternal age appeared to have a relatively minor effect, and the recurrence risk was mainly derived from other risk factors in this high‐risk population.

While overweight and obesity are strong risk factors for GDM, they are also the only modifiable risk factors for GDM that we could assess in the current study. A remarkable proportion (25%–45%) of women with GDM were of normal weight in our study. Moreover, most GDM diagnoses (~60%–70%) are based on only one abnormal OGTT value,^7^, ^14^, ^15^, ^22^, ^26^ as also shown in our study. From a public health perspective, women of normal weight with GDM and women diagnosed with GDM based on one abnormal OGTT value are also a large group with an increased risk of complications and morbidity during and after pregnancy.^27^ Moreover, GDM itself is a strong risk factor for T2D.^28^ Therefore, these groups should be taken into account when planning appropriate follow‐up and treatment during and after pregnancy.^10^, ^15^, ^27^, ^29^, ^30^, ^31^ For such women, other risk factors such as older age, genetic predisposition, poor diet, sedentary lifestyle, limited physical exercise, or lean obesity (characterized by a high body fat with normal BMI) may play the most crucial role in the development of GDM or its recurrence.^2^, ^3^, ^5^, ^6^, ^7^, ^24^, ^32^, ^33^ Further interventional studies are needed to test whether the number of abnormal OGTT values impacts the effectiveness of interventions to prevent subsequent GDM or long‐term morbidity, as well as to clarify the roles of modifiable factors, such as lifestyle, exercise habits, and weight management.

An important strength of this study is the use of a population‐based cohort from the Finnish MBR supplemented with numerical OGTT values from laboratory databases. It has been reported that the MBR has high quality and coverage.^34^ In addition, 99.7% of pregnant women in Finland attend public maternity clinics, where the same guidelines for pregnancy are used nationally.^35^ A major strength is the uniform national screening and diagnostic criteria for GDM that are still in use in Finland.^1^, ^10^, ^19^ A diagnosis of GDM as recorded in the MBR should be robust, as the accuracy of this diagnosis has been reported to be 94.3%.^19^ In addition, the OGTT results were collected from different regions of Finland from laboratories at secondary‐ and tertiary‐level delivery hospitals, which reduces the risk of selection bias. In our study, OGTT was performed, and glucose samples were analyzed in university and central hospital laboratories. A final strength of this work is that the rate of missing data for covariates was less than 2.1% for all important variables used in the analysis.

As a limitation of our study, we had no laboratory data for the OGTTs performed in the second pregnancy. Follow‐up data were based exclusively on the MBR data. In addition, a relatively small number of women had two or three abnormal OGTT values, which diminished the precision of the results. Moreover, the screening criteria were expanded from risk‐factor‐based to comprehensive screening in 2008, and the implementation of the recommendation was carried out during the same year. However, during the study period from 2009 to 2019, the national screening rates increased from 42% to 66%, and the incidence of identified GDM increased from 8.9% to 20.6%. This might be due to a learning curve to implement the new screening criteria or due to increases in risk factors for GDM, especially older age and obesity, among pregnant women.^36^, ^37^ Therefore, some women with moderate GDM risk factors might not have undergone the OGTT screening and were thus excluded from our study cohort. In this setting, we excluded multiparous and low‐risk women without an indication to undergo OGTT; as a result, the cohort members represent a primiparous high‐risk population.^1^ According to the Finnish guidelines, women with fasting glucose ≥7.0 mmol/L discontinued the OGTT.^1^, ^20^ We included only women with all three OGTT values in our study to provide the rationale for the study question based on the number of OGTT values. Therefore, the study represents women with fasting glucose <7 mmol/L, which is widely accepted as a threshold for pre‐pregnancy DM.^10^, ^38^ The MBR does not include the data regarding ethnicity, but the Finnish population is generally homogenous, with a relatively small amount of ethnic diversity in 2009. Postpartum OGTT is recommended after GDM, but we do not have data characterizing how this has been carried out. This increases the possibility of missed T2D diagnoses and cases misclassified as GDM in the second pregnancy.^39^ We also do not have data regarding women who have not undergone OGTT in the first pregnancy and thus are unable to calculate positive or negative predictive values for the number of abnormal OGTT values in the first pregnancy. Notably, this study is based on the Finnish CCG, where OGTT cut‐offs are slightly higher than those recommended by the International Association of Diabetes and Pregnancy Study Groups (IADPSG). When using the Finnish criteria, 30% fewer cases are classified as abnormal compared with when the IADPSG criteria are applied.^40^ No correction for multiple tests was performed to avoid excessively conservative interpretations, as parameters have strong correlations with each other. For the main outcome, namely GDM in the second pregnancy, the number of analyses is relatively small, and a correction for multiple testing is not necessarily needed. Instead, the confidence and credibility of the estimate can be evaluated from the confidence intervals, which in this case clearly show the effect of OGTT values on the recurrence of GDM.

## 5 CONCLUSION

According to this population‐based study, the overall rate of GDM recurrence in the second pregnancy was 56%. For those with two or three abnormal OGTT values, this recurrence rate was significantly higher, being double that for those with just one abnormal value. However, one abnormal value already increased the risk of developing GDM in the second pregnancy sixfold when compared with those with normal values in the OGTT. This information could be used in counseling and in targeting the follow‐up, prevention, and screening of GDM in subsequent pregnancies for these high‐risk women after the first GDM pregnancy.

## AUTHOR CONTRIBUTIONS

Conceptualization: M.V., E.Ka., H.L., and M.G. designed the Finnish Gestational Diabetes (FinnGeDi) study protocol. E.Ke., M.V., and J.P. were responsible for the planning of this particular study design. Data curation, THL (Finnish Institute for Health and Welfare); formal analysis, J.P.; funding acquisition for this particular study, J.P., and for the FinnGeDi study, M.V.; investigation, J.P.; methodology, E.Ke., J.P., and M.V.; project administration, E.Ke.; supervision, E.Ke. and M.V.; validation, E.Ke.; visualization, J.P. and E.Ke.; writing—original draft, J.P.; writing—review and editing, E.Ke. and M.V. All authors have read and agreed to the published version of the manuscript.

## FUNDING INFORMATION

This research received funding from the University of Oulu, the Päivikki and Sakari Sohlberg Foundation and Emil Aaltonen Foundation. The FinnGeDi study was funded by several institutions and foundations: the Academy of Finland, Diabetes Research Foundation, Foundation for Pediatric Research, Juho Vainio Foundation, Novo Nordisk Foundation, Signe and Ane Gyllenberg Foundation, Sigrid Jusélius Foundation, Yrjö Jahnsson Foundation, Finnish Medical Foundation, research funds from Oulu University Hospital (state grants), research funds from Helsinki University Hospital (state grants), Medical Research Center Oulu, and the Finnish Institute for Health and Welfare.

## CONFLICT OF INTEREST STATEMENT

The authors declare no conflicts of interest.

## ETHICS STATEMENT

According to Finnish legislation, informed consent is not needed for a register‐linked study if the participants are not contacted. The Ethics Committee of North Ostrobothnia Hospital District approved the study protocol (reference number 33/2008) on June 19, 2008. The study conformed to the European Medicines Agency guidelines for good clinical practice and the Declaration of Helsinki. The research privacy statement is available online: https://thl.fi/en/research‐and‐development/research‐and‐projects/the‐finnish‐gestational‐diabetes‐study‐finngedi‐.

## Supporting information


Table S1.

Table S2.

## Data Availability

Access to clinical data is regulated by ethics approvals and individual consent. Access to registry data is subject to permission from the registry authorities. For enquiries regarding possible collaboration, please contact FinnGeDi's principal investigator and study coordinator, Adjunct Professor Marja Vääräsmäki, MD, PhD [marja.vaarasmaki@oulu.fi] or Marja Vääräsmäki, Oulu University Hospital, Department of Obstetrics and Gynaecology, PO Box 23, 90029 OYS, Oulu, Finland.
