# Supplementary material for: The recurrence risk of gestational diabetes according to the number of abnormal values in the oral glucose tolerance test
Source: Acta Obstet Gynecol Scand. 2025 May 2;104(8):1452–62. doi: 10.1111/aogs.15148 (PMC12283174; doi:10.1111/aogs.15148)
Supplement: Supplementary file 1 — Table S1. [file AOGS-104-1452-s002.docx]

**Table S1.** Univariate logistic regression analyses for gestational diabetes (GDM) in the second pregnancy.

|  |  |  |  |  | | |  |
| --- | --- | --- | --- | --- | --- | --- | --- |
| GDM n = 331, reference n = 1346 | OR | (95% CI) |  | n | (%) | p-value |  |
| GDM status in 1^st^ pregnancy | 7.44 | 5.71 | 9.69 | 1677 | (100.0) | < 0.001 |  |
| Number of abnormal values in 1^st^ OGTT | 4.66 | 3.76 | 5.77 | 1677 | (100.0) | < 0.001 |  |
| *One abnormal vs normal* | 6.18 | 4.62 | 8.26 | 1596 | (95.2) | < 0.001 |  |
| *Two or three abnormal vs normal* | 13.77 | 8.35 | 22.71 | 1427 | (85.1) | < 0.001 |  |
| Age, 1^st^ pregnancy | 1.02 | 0.99 | 1.05 | 1677 | (100.0) | 0.164 |  |
| Age, 2^nd^ pregnancy | 1.04 | 1.01 | 1.06 | 1677 | (100.0) | 0.004 |  |
| Pre-pregnancy BMI, 1^st^ pregnancy | 1.13 | 1.10 | 1.16 | 1663 | (99.2) | < 0.001 |  |
| Pre-pregnancy BMI, 2^nd^ pregnancy | 1.13 | 1.11 | 1.16 | 1657 | (98.8) | < 0.001 |  |
| Pharmacological treatment, 1^st^ pregnancy | 10.47 | 3.78 | 29.00 | 1677 | (100.0) | < 0.001 |  |
| Birthweight > 2 SDs, 1^st^ pregnancy | 2.35 | 1.26 | 4.40 | 1677 | (100.0) | 0.008 |  |
| Time between deliveries | 1.26 | 0.98 | 1.62 | 1677 | (100.0) | 0.067 |  |
| BMI change between pregnancies, kg/m^2^ | 1.58 | 1.36 | 1.83 | 1643 | (98.0) | < 0.001 |  |
| *> 4* | 2.27 | 1.59 | 3.24 | 1643 | (98.0) | < 0.001 |  |
| *2.01 to 4* | 1.85 | 1.40 | 2.456 | 1643 | (98.0) | < 0.001 |  |
| *-2 to 2* | 0.51 | 0.40 | 0.65 | 1643 | (98.0) | < 0.001 |  |
| *-2.01 to -4* | 0.78 | 0.45 | 1.34 | 1643 | (98.0) | 0.369 |  |
| *< -4* | 0.72 | 0.27 | 1.92 | 1643 | (98.0) | 0.516 |  |
| *SES* | 1.10 | 0.97 | 1.24 | 1287 | (76.7) | 0.134 |  |
| *Upper-level clerical* | 0.71 | 0.52 | 0.99 | 1677 | (100.0) | 0.040 |  |
| *Lower-level clerical* | 0.96 | 0.75 | 1.23 | 1677 | (100.0) | 0.725 |  |
| *Manual workers* | 1.50 | 1.08 | 2.09 | 1677 | (100.0) | 0.016 |  |
| *Others* | 0.95 | 0.71 | 1.28 | 1677 | (100.0) | 0.745 |  |
| *Missing* | 1.10 | 0.84 | 1.44 | 1677 | (100.0) | 0.489 |  |

GDM, gestational diabetes; OGTT, oral glucose tolerance test; BMI, body mass index; SD, standard deviation; SES, socioeconomic status; OR, odds ratio; CI, confidence interval
