# Supplementary material for: The recurrence risk of gestational diabetes according to the number of abnormal values in the oral glucose tolerance test
Source: Acta Obstet Gynecol Scand. 2025 May 2;104(8):1452–62. doi: 10.1111/aogs.15148 (PMC12283174; doi:10.1111/aogs.15148)
Supplement: Supplementary file 2 — Table S2. [file AOGS-104-1452-s001.docx]

**Table S2.** Univariate subanalyses of gestational diabetes (GDM) in the second pregnancy among women with one, and two or three abnormal values in oral glucose tolerance test (OGTT) in the first pregnancy.

|  |  |  |  |  | | |  |
| --- | --- | --- | --- | --- | --- | --- | --- |
| Two or three abnormal values in first pregnancy’s OGTT n = 81,  one abnormal value n = 250 | OR | (95% CI) |  | n | (%) | P value |  |
| Two or three abnormal values in 1^st^ OGTT vs one abnormal value | 2.23 | 1.30 | 3.81 | 331 | (100.0) | 0.003 |  |
| Early GDM (≤ 20 gestational weeks) | 1.02 | 0.48 | 2.18 | 331 | (100.0) | 0.956 |  |
| Abnormal OGTT values |  |  |  |  |  |  |  |
| *Fasting value* | 1.94 | 1.25 | 3.00 | 331 | (100.0) | 0.003 |  |
| *1-h value* | 1.13 | 1.00 | 1.27 | 331 | (100.0) | 0.057 |  |
| *2-h value* | 1.11 | 0.97 | 1.27 | 331 | (100.0) | 0.141 |  |
| Age, 1^st^ pregnancy | 1.01 | 0.96 | 1.05 | 331 | (100.0) | 0.844 |  |
| Age, 2^nd^ pregnancy | 1.02 | 0.97 | 1.07 | 331 | (100.0) | 0.426 |  |
| Pre-pregnancy BMI, 1^st^ pregnancy | 1.11 | 1.06 | 1.16 | 329 | (99.4) | < 0.001 |  |
| Pre-pregnancy BMI, 2^nd^ pregnancy | 1.13 | 1.08 | 1.18 | 328 | (99.1) | < 0.001 |  |
| Pharmacological treatment, 1^st^ pregnancy | 2.46 | 0.87 | 6.93 | 331 | (100.0) | 0.089 |  |
| Birthweight > 2 SD, 1^st^ pregnancy | 3.64 | 0.77 | 17.09 | 331 | (100.0) | 0.102 |  |
| Interdelivery interval ≤ 24 or > 24 months | 0.90 | 0.57 | 1.43 | 331 | (100.0) | 0.657 |  |
| BMI change between pregnancies, kg/m^2^ | 1.53 | 1.16 | 2.03 | 326 | (98.5) | 0.003 |  |
| *> 4* | 3.82 | 1.41 | 10.36 | 326 | (98.5) | 0.008 |  |
| *2.01 to 4* | 1.32 | 0.75 | 2.32 | 326 | (98.5) | 0.337 |  |
| *-2 to 2* | 0.52 | 0.14 | 1.87 | 326 | (98.5) | 0.314 |  |
| *-2.01 to -4* | 0.70 | 0.45 | 1.11 | 326 | (98.5) | 0.128 |  |
| *< -4* | 0.71 | 0.33 | 1.57 | 326 | (98.5) | 0.403 |  |
| *SES* | 0.94 | 0.74 | 1.21 | 331 | (100.0) | 0.648 |  |
| *Upper-level clerical* | 0.96 | 0.52 | 1.79 | 331 | (100.0) | 0.896 |  |
| *Lower-level clerical* | 1.11 | 0.69 | 1.79 | 331 | (100.0) | 0.663 |  |
| *Manual workers* | 0.98 | 0.54 | 1.78 | 331 | (100.0) | 0.946 |  |
| *Others* | 0.85 | 0.47 | 1.51 | 331 | (100.0) | 0.571 |  |
| *Missing* | 1.05 | 0.63 | 1.76 | 331 | (100.0) | 0.848 |  |

GDM, gestational diabetes; OGTT, oral glucose tolerance test; BMI, body mass index; SD, standard deviation; SES, socioeconomic status; OR, odds ratio; CI, confidence interval
